# Dissociation of Modular Total Hip Arthroplasty at the Neck-stem Interface: A Unique but Possible Complication

**DOI:** 10.7759/cureus.5556

**Published:** 2019-09-02

**Authors:** Angelos Trellopoulos, Stavros Angelis, George Komnos, Grigorios Avramidis, Euaggelos Gikas

**Affiliations:** 1 3rd Orthopaedic Department, Hygeia Hospital, Athens, GRC; 2 Orthopaedics, General Hospital Hellenic Red Cross Korgialenio - Benakio, Athens, GRC; 3 Orthopaedics, General University Hospital of Larissa, Larissa, GRC; 4 Orthopaedics, Chalkida General Hospital, Chalkida, GRC

**Keywords:** total hip arthroplasty, modular hip, components dissociation, open reduction

## Abstract

Mechanical failure of total hip arthroplasty (THA) is often related to dislocation of the hip. In hip arthroplasty with a dual-modularity prosthesis, the surgeon has to face the unique disadvantage of the dissociation of its components. Most cases reported are related to the dissociation of the neck-head interface and only an extremely small percentage is due to dissociation at the neck-stem interface. We report a case with dissociation at the neck-stem interface generated by a fall. Possible reasons for dissociation of the modular system are presented. An open reduction using the same neck system was performed. We suggest that surgeons should be aware of this particular problem, which is related to the nature of the system.

## Introduction

Total hip arthroplasty (THA) is a successful procedure for the treatment and restoration of the function of the hip joint [1]. Since the early 1970s, modular connection for hip prosthesis has been used for heads with different neck sizes or diameters [1]. Modular neck adapters were introduced in the mid-1990s [2-3]. The modular femoral components provide a variety of advantages, the most important of which is the ability to select the head and neck size intraoperatively [4]. The orientation of the neck, which is one of the main causes of postoperative dislocation of the hip, can also be altered after the implantation of the stem. Dislocation of the modular THA after a fall is common [5]. Dissociation at the neck-stem interface without hip dislocation is extremely rare. To our knowledge, only one nontraumatic case has been described [6]. We report a case of the neck-stem interface dissociation after falling of own height. The cause of dissociation and its treatment are discussed. 

## Case presentation

A 77-year-old female underwent a THA of her right hip in our hospital in 2007 because of degenerative arthritis. A posterior approach was performed. A modular THA system was placed (Encore Medical LP DJO Surgical, Dalla, TX) and the material of the prosthesis was cobalt-chrome alloy. The hemispherical porous acetabular component (52 mm diameter) was fixed with the press-fit technique. The polyethylene liner was group 2, 10°, 28 mm in the inner diameter. With regard to the femoral component, a modular femoral stem (LSF series) porous-coated was fixed. The patient had an uneventful postoperative period and mobilization started the third day after the surgery. She was discharged seven days later, with instructions to partial weight bear for three weeks with the support of an orthopedic walker.

**Figure 1:**
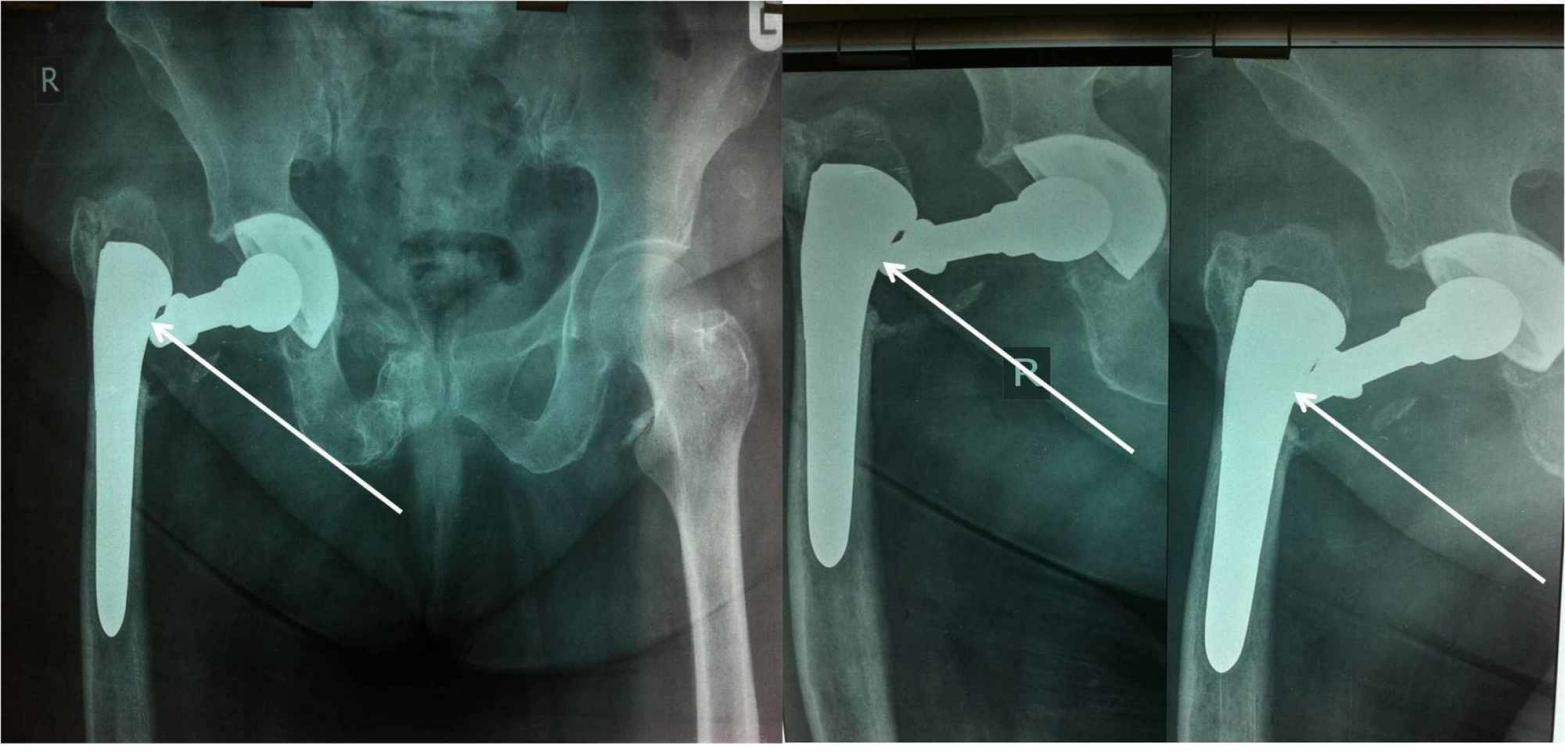
Radiographs show the dissociation of the femoral neck-stem interface after the fall (white arrows).

The radiographic follow-up during the first and the third month indicated no problem and the patient was counseled to walk without restrictions. Eight years postoperatively, she presented to our department reporting a fall of own height three months ago. She was unable to bear weight and walking without support was impossible. She complained of a very painful operated hip. Radiographs showed dissociation between the stem and the neck interface without dislocation of the head (Figure 1). A complete laboratory testing was performed and infection was ruled out.

**Figure 2:**
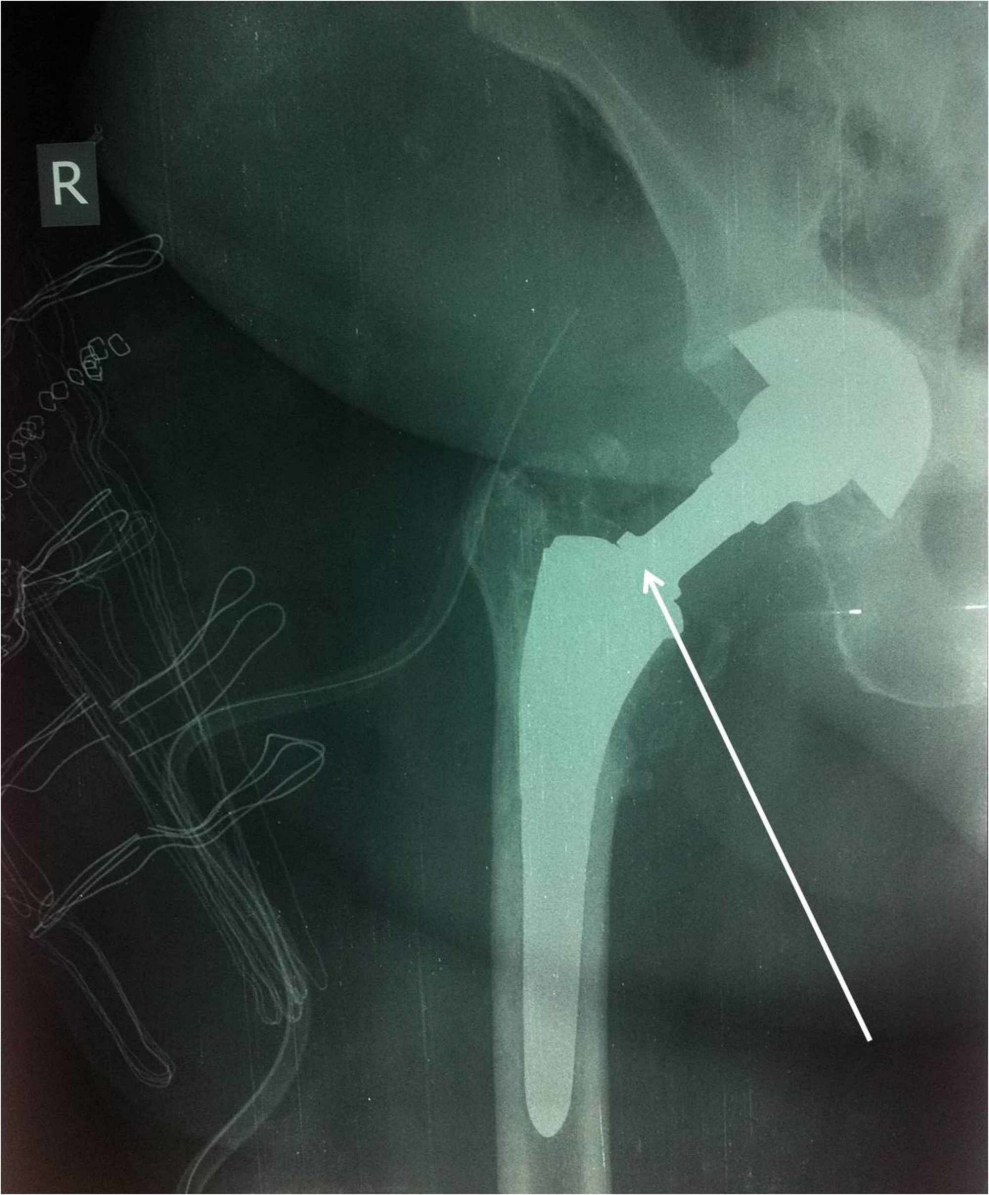
Radiograph after open reduction. Stability test in the neck-stem junction (white arrow) of the modular stem revealed that the prosthesis was stable.

Subsequently, an open reduction was decided as the appropriate treatment. The same posterior approach was utilized and the same neck system was used. The stability test in the neck-stem junction of the modular stem revealed that the prosthesis was stable (Figure 2). Radiographs that were obtained during the first and six months postoperatively showed no signs of instability of the stem. Additionally, the patient declared satisfied with the function of her operated hip.

## Discussion

Modular THA holds the advantages of choosing the angle, offset, and femoral anteversion intraoperatively. On the other hand, there is surface micromotion between the compartment and the junctions of the modular system [7]. This micromotion can lead to mechanical failure. One of the largest clinical studies for modular THA by Grupp et al. revealed that it is in the mechanical nature of the modular system to cause fretting between its components, due to micromotion [8]. This is an unavoidable side-effect of modularity. In this fretting zone, microcracks develop, leading to dynamic fatigue fracture of the implant. In this study, 87% of cases fretting were accompanied by crevice corrosion. The combination of those two can generate microcracks and can accelerate implant failure.

Micromotion and fretting of the stem-neck interface have also been reported in several studies as a cause of inflammatory tissue reaction through the possible generation of metal ions inside the modular coupling. The excessive absorption of this metal debris can lead to bone resorption and increased inflammation leading to instability or a catastrophic fracture [9-10].

The orientation of the femoral neck plays a significant role in the stability of the neck stem interface [11]. In combination with the orientation of the acetabular cup, head-neck ratio, and the design of the acetabular opening, it sets the stable range of motion (ROM) of the hip. Malpositioning of the neck orientation has a major impact on ROM, increasing the risk of impingement between the neck of the femoral component and the rim of the acetabular cup. This leads to micromotion of the acetabular cup and implant loosening. If more mechanical stress is applied, then dissociation of the components or joint dislocation may be produced.

Heterotopic ossification (HO) after THA is another factor leading to abnormal movement of the hip. HO represents one of the most common complications after THA with published rates ranging from 5% to 90% depending on the risk factors [12]. Several studies reveal that factors such as sex, history of HO after previous hip surgery, hypertrophic osteoarthritis, type of anaesthesia, even surgical approach, trauma and other factors can play a significant role for the appearance of this phenomenon [13]. The heterotopic ossification after THA varies from small bone spurs to complete ankylosis of the hip joint [14]. The ectopic bone reduces the hip joint ROM. In a modular system apart from the mechanical failure of the junction, this can cause head dislocation [15].

## Conclusions

Modular THA system has advantages and disadvantages, as all implants do. It has the additional benefit of intraoperative orientation choice, but fretting or crevice corrosion may lead to mechanical failure. Dissociation at the neck-stem interface without hip dislocation is extremely rare. We suggest that surgeons should be aware of this particular problem, which is related to the nature of the system.
